# Change-of-Direction Deficit and Positional Physical Profiles in Youth Futsal Players: A Cross-Sectional Study

**DOI:** 10.3390/sports13080263

**Published:** 2025-08-12

**Authors:** Oscar Villanueva-Guerrero, Oliver Gonzalo-Skok, Rafael Albalad-Aiguabella, Elena Mainer-Pardos

**Affiliations:** 1 Health Sciences Faculty, Universidad San Jorge, Autovía A23 km 299, Villanueva de Gállego, 50830 Zaragoza, Spain; ovillanueva@usj.es (O.V.-G.); ralbalad@usj.es (R.A.-A.); 2 Department of Communication and Education, Universidad Loyola Andalucía, 41704 Seville, Spain

**Keywords:** team sports, performance, body composition, interlimb asymmetry, exercise testing, biomechanics

## Abstract

This study aimed to describe and assess differences among playing positions, to determine playing position profiles, and to analyze the relationships between the change-of-direction deficit (CODD) percentage and the other anthropometric and performance variables. A total of 98 young futsal players (age: 17 ± 1 years) from the highest national level in Spain were assessed using a cross-sectional design. Anthropometric variables such as height and body mass were recorded. The performance tests included countermovement jumps; horizontal jumps; sprint tests (10 m and 25 m); change-of-direction (COD) tests, including a 10 m test with one COD of 180° (COD180) and a 25 m test with 4 CODs (V-cut); and the percentage CODD. Furthermore, asymmetries were recorded. The group comparisons were considered statistically significant at *p* ≤ 0.05 and were supported by the effect sizes and mean differences. Significant differences were found among playing positions, showing that pivots and goalkeepers were significantly taller than left- and right-wingers and defenders (*p* < 0.05, effect size (ES) = −1.42 to 0.72). Goalkeepers were significantly slower than the rest of the positions in COD180 to the left (*p* < 0.05, ES = 1.32 to 1.89). A very large association was found between the CODDs of 25 m and 25 m (*p* < 0.001; r = −0.72). These results suggest that pivots and goalkeepers are taller and larger than the other players. However, aside from goalkeepers, no differences in performance variables were observed among the outfield players. In addition, a lower %CODD is associated with a faster COD performance, highlighting its importance in training.

## 1. Introduction

Futsal is a high-intensity, intermittent sport that demands a combination of technical, tactical, and physical attributes from its players [1]. The game is played on a court measuring 40 m × 20 m, with 3 m × 2 m goals, and consists of two teams of five players, including a goalkeeper [2]. Matches are played in two 20 min halves with stopped time, and substitutions are unlimited, allowing continuous player rotation [3]. Due to the confined playing space and rapid transitions, futsal requires players to possess exceptional change-of-direction (COD) abilities, speed, and power to maintain a high performance throughout the match [4].

Recent studies have examined the fitness profiles of futsal players across different competitive levels and genders, highlighting their anthropometric characteristics and physical attributes such as their jump performance (CMJ height: 25.7 cm to 37.7 cm), sprint times (10 m: 1.74 s to 1.98 s), and COD ability (505 COD: 2.81 s to 2.91 s), which are commonly associated with a higher competitive performance [5,6,7]. Although not significantly different between senior and junior futsal players, anthropometric variables show that seniors tend to have a greater lean body mass, likely due to prolonged exposure to systematic training, which contributes to an improved physical performance [7]. However, an excessive body fat percentage has been negatively correlated with futsal-specific agility and reactive strength (*p* < 0.01), highlighting its detrimental impact on high-intensity movements such as sprinting, jumping, and rapid CODs [8]. In terms of physical capacities, Sekulic et al. showed that top-level players outperform high-level players in key fitness attributes such as their reactive strength index (163 index vs. 140 index), broad jump (248 cm vs. 235 cm), and kicking speed (96–108 km/h vs. 91–104 km/h), as well as futsal-specific reactive agility, particularly when dribbling the ball (2.52 s vs. 2.66 s) (*p* < 0.05) [8]. Moreover, differences between elite and sub-elite players have been identified, particularly in aerobic fitness, acceleration, and strength levels, which are determinants for performance at higher levels of competition [9]. Importantly, comparisons among playing positions have also revealed distinct anthropometric and physical demands. Gadea-Uribarri et al. (2025) observed that pivots accumulated the lowest external load values across a competitive microcycle, while wings and defenders experienced the highest values in variables such as accelerations and decelerations [10]. Goalkeepers and pivots often present a higher body mass and lower acceleration demands due to their tactical roles [5]. These position-specific differences highlight the relevance of analyzing physical profiles by playing position in order to individualize training interventions and performance monitoring [11]. To the best of our knowledge, no study has specifically analyzed the anthropometric and performance profiles of young male futsal players by playing position, limiting the current understanding of their developmental needs, position-specific physical demands, and implications for individualized training and injury prevention strategies.

One of futsal’s most critical performance determinants is the ability to execute rapid COD maneuvers, as players frequently adjust their positioning in response to dynamic game situations [12]. Given the high frequency of COD actions in futsal, optimizing this ability is essential for improving movement efficiency and game performance. Studies have shown that futsal players are generally more efficient at changing direction than soccer players (ES = 1.11), though they exhibit similar COD performance metrics to other team-sport athletes, such as rugby and handball players (*p* > 0.05) [13]. To better quantify COD ability, the COD deficit (CODD) concept has been introduced as an alternative to traditional time-based measures. The CODD is the difference between the time taken to complete a specific distance, including a COD maneuver, and the time required to sprint the same distance in a straight line [14]. More recently, the CODD percentage (%CODD) has been proposed as a normalized metric, providing a percentage-based perspective of this difference, which allows for a more accurate evaluation of an athlete’s COD efficiency relative to their linear speed [15]. This approach mitigates potential misinterpretations when comparing athletes with different sprinting abilities, making the %CODD a more relevant metric for coaches and practitioners. Despite its increasing application in soccer, no study is currently analyzing the relationship between the CODD and performance variables in futsal.

Despite the growing literature on futsal performance [5,16,17], limited research has been conducted on the young male futsal population. Understanding young futsal players’ anthropometric and performance profiles is essential for optimizing training programs, identifying talent, and implementing evidence-based injury prevention strategies. Most studies have focused on senior or elite-level athletes [5,16,18,19], overlooking the developmental characteristics of younger populations, whose physical and neuromuscular adaptations may differ significantly due to growth, maturation, and training exposure. This is particularly relevant in youth players, whose bodies are still adapting physically and neuromuscularly to training demands. During this stage, mismatches in the development of bones, muscles, and tendons may contribute to asymmetries, flexibility limitations, or reduced motor control [20,21], ultimately increasing their susceptibility to musculoskeletal overuse injuries [22]. Monitoring biomechanical variables such as interlimb asymmetries and the CODD from an early stage may help anticipate the injury risk and guide preventive strategies tailored to individual maturation profiles [23]. Notably, asymmetries exceeding 15% in the unilateral CMJ height have been prospectively linked to an increased incidence of lower-limb injuries in youth athletes [23,24,25].

Furthermore, although the change-of-direction deficit (CODD) has been widely examined in soccer, its applicability and relevance in futsal remain unclear, given the sport’s unique movement demands and court dimensions. Without a clear understanding of how the CODD interacts with key performance determinants in young futsal players, coaches and practitioners may lack the necessary insights to develop effective training interventions that enhance the CODD efficiency and overall performance. Similarly, interlimb asymmetries, frequently associated with an increased risk of injury, remain understudied in youth futsal, despite their potential impact on movement efficiency and physical balance. It is hypothesized that physical and anthropometric characteristics differ across playing positions, and that both CODD and inter-limb asymmetries show significant relationships with selected physical performance variables. Therefore, the current study aimed to assess playing position differences, determine playing position profiles, and analyze the relationships among the %CODD, interlimb asymmetries, and the remaining anthropometric and performance variables.

## 2. Materials and Methods

### 2.1. Participants

This study included ninety-eight highly-trained young futsal players (17 ± 1) (TIER 3) [26]. A preliminary power analysis was conducted to determine the number of participants using the GPower software (version 3.1.9.3, based in Düsseldorf, Germany). A one-way ANOVA with five groups was used, considering an effect size of 0.5, a significance level (alpha) of 0.05, and a statistical power of 95%. The analysis determined that 80 subjects were required; our final sample exceeded this requirement, ensuring a greater robustness in the analysis. The local ethics committee approved the study (C.P.–C.I PI24/137, act n°07/2024, CEICA, Zaragoza, Spain) and followed the Declaration of Helsinki. The participants signed an informed consent form, and for underage players, their parents or legal guardians also signed the data consent form.

### 2.2. Study Design

This study investigated the physical characteristics of young futsal players from eight teams competing at Spain’s highest national young level (Young Honor Division Group VI). They followed a standardized weekly training schedule consisting of three futsal-specific sessions (each lasting 90 min) and one official match per week (40 min of stopped-clock time), totaling 4.5 h of training. The training methodology and volume were consistent across all teams, and all players actively competed during the data collection period. The inclusion criteria were as follows: (1) an age between 17 and 19 years; (2) participation in the highest national futsal category; and (3) no injuries in the month before testing. The exclusion criteria included the following: (1) not being an active team player and (2) suffering an injury during the month prior to the evaluations. The participants were grouped according to their on-field positions, which constituted the main independent variable of the study: goalkeeper (*n* = 15), defender (*n* = 22), winger—left (*n* = 16), winger—right (*n* = 27), and pivot (*n* = 18). In total, 104 players were assessed for eligibility. Of these, 6 were excluded due to recent injuries, leaving a final sample of 98 participants who met all criteria and completed all assessments. A descriptive analysis was carried out to define and characterize the physical profile of these athletes. To this end, a cross-sectional design was used to systematically evaluate the differences in physical variables among players of different playing positions. The relationship between the percentage change in the directional deficit and the other anthropometric and performance variables was also analyzed.

To obtain a more detailed picture of the physical profile of the participants, 98 players were assessed through a series of anthropometric measurements and physical performance tests. The anthropometric assessments included height, body mass, and a body composition analysis. On the other hand, physical performance was assessed through specific tests, such as the bilateral and unilateral countermovement jump (CMJ) tests, the bilateral and unilateral horizontal jump (HJ) tests, the 10 m and 25 m sprint tests, the 180° change-of-direction test (COD180). and the V-cut test.

### 2.3. Procedures

To reduce the risk of fatigue, the participants were instructed to refrain from strenuous physical activity for 48 h prior to testing. They were also reminded of the importance of maintaining proper nutrition and hydration throughout this period to ensure optimal testing conditions. Furthermore, as all participants had previously performed these assessments on a minimum of five occasions, they were well-acquainted with the testing procedures, thereby minimizing potential learning effects. Prior to the assessments, a dynamic warm-up aligned with the RAMP framework (raise, activate, mobilize, and potentiate) was conducted [27]. The tests were conducted on an indoor futsal court using players’ own futsal-specific footwear. The same lead researcher conducted and supervised all assessments to ensure consistency of data collection. The order of execution was the bilateral and unilateral CMJs, the bilateral and unilateral HJs, the sprint tests (10 m and 25 m), the COD180, and the V-cut test. All technologies used for data collection have been previously validated and included the MyJump2 app for a vertical jump assessment and Witty photocell timing gates (Microgate, Bolzano, Italy) for the sprint and COD tests. Reliability was ensured through intra-rater consistency, with high intraclass correlation coefficients (ICC = 0.89 to 0.98) and low coefficients of variation (CV = 0.6 to 5.7%) across all tests, as detailed in each subsection.

#### 2.3.1. Bilateral and Unilateral Countermovement Jumps

The participants performed the countermovement jump (CMJ) tests with their hands on their hips to prevent arm swing. A countermovement was allowed before take-off, ensuring full extension of the hip, knee, and ankle during the flight phase. The testing sequence began with the bilateral CMJ, in which two trials were performed, with 45 s of rest between attempts. The best result was recorded for the analysis. Following this, the unilateral CMJ was performed, first with the left leg and then with the right leg. In this test, the participants jumped on one leg while keeping the opposite leg flexed at 90° at the hip and knee, avoiding any swinging or balance-assisting movement. The best value for each leg was recorded to assess the interlimb asymmetry. A trial was considered invalid if the participant failed to maintain proper posture or did not land on the same leg.

All jumps were analyzed using an iPhone 12 and the MyJump2 app, a scientifically validated and reliable application [28,29] that calculates the jump height based on the flight time. To ensure methodological consistency, all CMJ data were collected and analyzed by the same researcher. The intraclass correlation coefficient (ICC) was 0.90–0.98, and the coefficient of variation (CV) was 1.7–5.7%. The calculation of interlimb asymmetry was performed using the following equation [30]:

Interlimb asymmetry = 100/Max Value (right and left) × Min Value (right and left) × −1 + 100.(1)

#### 2.3.2. Bilateral and Unilateral Horizontal Jumps

The HJ test was conducted from a static position using both legs. The participants were required to land stably without losing balance. The use of arms was allowed during this test [31]. The HJ performance was assessed using a standard tape measure to record the distance covered. The total distance covered was recorded. Two valid attempts were recorded for each leg, with 45 s of rest between jumps, and the best result was used for further analysis. The testing sequence always began with the bilateral HJ, followed by the unilateral HJ with the left leg, and then with the right leg. A unilateral HJ was also performed. The participants were allowed to use arm swing and their free leg for propulsion, ensuring a stable landing on the jumping leg. Interlimb asymmetry was calculated using the formula by Bishop et al. [30]. The ICC values were 0.91–0.93 and the CVs were 1.7–2%. The participants performed the test with both legs while keeping their hands on their hips to prevent arm movement. A countermovement was allowed before take-off, ensuring the full extension of the hip, knee, and ankle during the jump. Two trials were performed, with 45 s of rest.

#### 2.3.3. 10 m and 25 m Sprint Test

The sprint speed was evaluated with a 25 m straight sprint test, which also provided a 10 m split time. The sprint times were measured using a dual-beam photocell system (Witty, Microgate, Bolzano, Italy). The participants started from a 2-point staggered stance, positioning their lead foot 0.5 m behind the initial timing gate. The photocells were set at a height of 0.75 m and spaced 1.5 m apart. Each participant completed the 25 m sprint twice, with a minimum of 3 min of passive rest between attempts. The fastest trial was retained for the analysis. The reliabilities were 0.89–0.95 and 0.8–1.3% for the ICCs and CVs, respectively.

#### 2.3.4. Change of Direction—180° and COD Deficit

A 10 m shuttle run with a 180° direction change was used to assess the COD ability (COD180 test). The players started in a two-point staggered stance, 0.5 m behind the first timing gate (0 m). They sprinted 5 m forward, planted and turned 180° off one leg, and sprinted 5 m back to the start/finish line. Two trials were completed using a turn off the left leg and two off the right leg, in alternating order. There was a 2 min rest between each trial. The fastest trial for each turning leg was recorded, yielding two performance metrics: COD180L (turn off the left leg) and COD180R (turn off the right leg). Timing gates (Witty, Microgate) at the start/finish captured the total time. The test reliability was high (ICC = 0.91–0.93; CV = 1.1–1.5%). The interlimb asymmetry was calculated using the equation proposed by Bishop et al. [30]. In addition, the change-of-direction deficit for the 180° test was computed as a percentage, following Nimphius et al. [14], to quantify the performance decrement due to the direction change. The percentage COD deficit for each leg was calculated using the following formula:

((COD time − 10 m sprint time)/10 m sprint time) × 100(2)

#### 2.3.5. V-Cut Test and COD Deficit 25

To assess the multidirectional agility, a 25 m V-cut test was performed, in which the players executed four 45° directional changes spaced every 5 m. The test started from a two-point staggered stance, with the front foot positioned 0.5 m behind the timing system. Photocells (Witty, Microgate, Bolzano, Italy) were placed at both the starting and finishing lines, set at a height of 0.75 m and spaced 1.5 m apart. For a trial to be valid, the participants were required to step entirely over a marked floor line with at least one foot at each change of direction. Attempts not meeting this criterion were repeated. Each athlete completed two valid trials with three minutes of passive recovery between them, and the fastest time was used for the analysis. The test demonstrated an excellent reliability, with ICC values of 0.95 and a coefficient of variation (CV) of 0.6%. The change-of-direction deficit was calculated as a percentage using the standard equation previously described in the literature [14]:

((V-cut time − 25 m sprint time)/25 m sprint time) × 100(3)

### 2.4. Statical Analysis

All data were analyzed using IBM SPSS Statistics (version 25, IBM, New York, NY, USA) and Microsoft Excel (version 2016, Microsoft Corp., Redmond, WA, USA). Descriptive statistics are reported as the mean ± standard deviation (SD). The within-session reliability was assessed using the coefficients of variation (CVs) and intraclass correlation coefficients (ICCs), calculated through a spreadsheet specifically designed for this purpose. The distribution of the data was assessed using the Kolmogorov–Smirnov test. All variables met the assumption of normality except for the interlimb asymmetries in the COD and CODD. Levene’s test was used to evaluate the homogeneity of variances (homoscedasticity). To examine differences across playing positions (left-wingers, right-wingers, pivots, defenders, and goalkeepers), a one-way analysis of variance (ANOVA) was conducted. When significant main effects were found (*p* < 0.05), Bonferroni’s post hoc test was used to identify specific pairwise differences. For variables not fulfilling the assumptions of normality—specifically, the interlimb asymmetries in the COD and CODD—a nonparametric Kruskal–Wallis test was employed (*p* < 0.05). Effect sizes for the ANOVA were calculated using eta-squared (η^2^), with the thresholds interpreted as small (>0.01), medium (>0.06), and large (>0.14). Cohen’s d was also computed based on the pooled standard deviation to determine the magnitude of positional differences, with cut-offs set at >0.2 (small), >0.5 (moderate), and >0.8 (large). To explore associations between physical performance variables and the percentage-based change-of-direction deficits (CODD) for the right and left legs, as well as the V-cut test, Pearson’s correlation coefficients (r) were calculated. The strength of these correlations was interpreted using the following scale: ≤0.1 (trivial), >0.1–0.3 (small), >0.3–0.5 (moderate), >0.5–0.7 (large), >0.7–0.9 (very large), and >0.9–1.0 (almost perfect) [32]. Confidence intervals at 95% (95% CI) were reported for both the effect sizes and the correlation coefficients.

## 3. Results

The descriptive data of the players are provided in Table 1.

The analysis of variance revealed significant differences among playing positions for several variables. Regarding the anthropometric characteristics, differences were found for the age (F = 3.95, *p* < 0.05, η^2^ = 0.14 [95% CI: 0.02–0.25]), height (F = 6.62, *p* < 0.001, η^2^ = 0.22 [0.07–0.33]), and body mass (F = 8.99, *p* < 0.001, η^2^ = 0.28 [0.11–0.39]). The post hoc comparisons indicated that pivots and goalkeepers were significantly taller than left- and right-wingers and defenders (*p* < 0.05, ES = −1.42 to 0.72), while goalkeepers had a significantly higher body mass than all the other outfield positions (*p* < 0.05, ES = −1.49 to 0.95). In addition, pivots were significantly heavier than right-wingers (*p* < 0.05, ES = −1.28).

In performance-related variables, significant differences were observed for the CODL (F = 8.36, *p* < 0.001, η^2^ = 0.26 [0.09–0.38]), CODR (F = 2.64, *p* = 0.039, η^2^ = 0.10 [0.00–0.19]), and V-cut test (F = 3.21, *p* = 0.016, η^2^ = 0.12 [0.00–0.24]). Specifically, left-wingers performed significantly better than goalkeepers for both the CODR and V-cut (*p* < 0.05, ES = 1.05 to 1.22), while goalkeepers were significantly slower than all other positions for CODL (*p* < 0.05, ES = 1.32 to 1.89).

**Figure 1:**
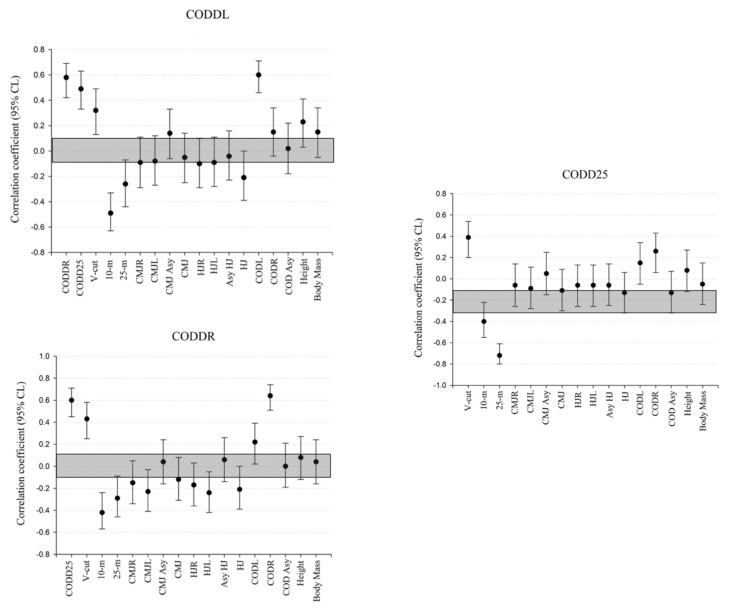
Correlation coefficients (95% confidence interval) describing the relationships between the percentage-based change-of-direction deficit, for both left (CODDL) and right (CODDR), and the V-cut (CODD25) with physical performance and anthropometric variables. CMJR and CMJL: unilateral countermovement jump with right and left legs; HJR and HJL: unilateral horizontal jump with right and left legs; COD180R and COD180L: 10 m shuttle sprint with one change of direction to right or left; CMJ: bilateral countermovement jump; HJ: bilateral horizontal jump; 10 m: linear sprint of 10 m; 25 m: linear sprint of 25 m; CODR and CODL: 10 m shuttle sprint with one change of direction of 180° to right or left; CODDL, CODDR, and CODD25: change-of-direction deficit with right or left and V-cut; Asy: asymmetry.

In terms of the relationships between the change-of-direction deficit variables and performance outcomes (Figure 1), CODDL showed moderate correlations with the CODD25, the V-cut, and the 10 m sprint times (*p* < 0.05; r = −0.49 to 0.49), and large correlations with the CODL and CODDR (*p* < 0.001; r = 0.58 to 0.60). The CODDR was moderately associated with the V-cut and 10 m (*p* < 0.001; r = −0.42 to 0.43) and strongly correlated with the CODD25 and CODR (*p* < 0.001; r = 0.60 to 0.64). The CODD25 showed moderate relationships with both the V-cut and 10 m (*p* < 0.001; r = −0.40 to 0.39), and a very large correlation with the 25 m sprint (*p* < 0.001; r = −0.72).

## 4. Discussion

The current study aimed to assess the differences among playing positions, to determine playing position profiles, and to analyze the relationships between the % CODD and the rest of the anthropometric and performance variables. The main findings were as follows: (1) pivots and goalkeepers are taller and bigger than the rest of the positions; (2) all playing positions have similar profiles in jumping, linear and multidirectional sprinting ability, and interlimb asymmetries except for goalkeepers; (3) the change-of-direction speed measured through single and multiple changes of direction is worse for goalkeepers than for all other playing positions; and (4) a lower CODD percentage seems to be affected by a faster COD speed. These results suggest that interlimb asymmetries may not differ substantially among the positions in elite youth futsal, although their presence continues to be a relevant factor for performance and injury risk monitoring in applied settings.

The anthropometric profiles of young futsal players show that pivots and goalkeepers have a greater height and body mass than other positions. This aligns with previous studies on elite male players [33,34] and elite Spanish female futsal players [35]. Ramos et al. also found that goalkeepers and forwards had a higher body mass than wingers and defenders [36], indicating that anthropometric characteristics may influence role allocation within the team and the physical demands of each position [35]. In addition to these morphological differences, pivot players appear to be exposed to a lower total external load than their teammates, which may influence their training and physical preparation requirements [11,37]. This lower external demand may be related to their role in the game, which is characterized by more specific and controlled actions compared to positions that require a higher volume of movement and frequent changes of pace [10]. However, the results are not entirely consistent in all the populations studied. Albalad-Aiguabella et al. found no significant differences among the playing positions for female futsal players (*p* > 0.05) [5], indicating that factors such as gender, level of competition, or tactical strategies may influence these variations. Characterizing these physical profiles will allow for optimized training planning, facilitating more specific approaches tailored to the actual demands of each position in futsal.

Several previous studies in futsal players have analyzed performance variables through different training programs, examining their impact on the development of physical abilities such as strength and speed [38,39,40,41,42]. Regarding the CMJ variable, our results (35.9 ± 3.99 cm) are consistent with those described in the literature, which range between 35.5 and 38.7 cm [39,43,44]. Spyrou et al. recently explored the CMJ performance across playing positions in elite futsal and found values ranging from 35.1 cm in defenders to 37.3 cm in wingers, although no significant differences (*p* > 0.05) were observed among positions, supporting the notion that positional differences may exist, but remain within a small range [17]. In the 10 m sprint, our players recorded an average time of 1.89 ± 0.07 s, aligning with previous research findings, which have reported values ranging from 1.68 to 2.23 s [43,44,45]. Similarly, in the 25 m sprint, our results (3.84 ± 0.14 s) are comparable to those reported in another study, which found values ranging from 3.76 to 3.83 s [40]. Ayarra et al. found no significant differences across competitive levels in acceleration over 5 and 15 m or in the 505 agility test; however, players from higher-level categories performed better in the horizontal and vertical jump tests, suggesting that explosive power may serve as a discriminating factor based on competitive level [2]. These findings indicate that the physical profile of our sample is representative of highly trained young futsal players, which strengthens the external validity of the results. Notably, the only study that has compared performance variables by playing position in futsal is that of Albalad-Aiguabella et al., whose results closely resemble those found in the present study [5]. In this study, no significant differences were observed among the field positions, which can be explained by the homogeneous training loads and the lack of specialization at these ages [46]. However, goalkeepers showed a clearly differentiated profile, possibly due to the specific demands and nature of their position [47]. Even when standard physical tests do not show large differences among positions, the functional demands during gameplay do vary and justify a position-specific training periodization [48]. Therefore, more studies are needed to establish well-defined positional profiles and to standardize performance variables in futsal, allowing for more precise comparisons and practical applications in training and talent identification.

In futsal, the COD speed is one of the most decisive actions due to the rapid changes of activity during the match [49]. Outfield players must move quickly and accurately to keep or regain possession of the ball, which requires high linear and multidirectional sprinting abilities [50]. In our study, goalkeepers showed a lower COD speed compared to outfielders, which may be justified by differences in the physical and functional demands of their position. While outfielders perform constant CODs at multiple angles, goalkeepers rely more heavily on explosive lateral movements in a frontal plane, which may limit their development in this ability [5]. Freitas et al. indicated that athletes with a greater strength and power tend to perform CODs with a greater speed and efficiency [51]. In this sense, a lower CODD percentage indicates that the loss of speed when changing direction is lower compared to the speed achieved in a linear sprint, reflecting a higher efficiency in this action [52]. Players with a lower CODD will be able to change direction more quickly and smoothly without sacrificing speed [14]. However, despite the high relationship observed between the CODD and COD performance, no significant differences were found among positions. This can be explained by the fact that the CODD represents a relative and individual measure of efficiency in change of direction, and not an absolute measure of performance. Therefore, even if one player is faster than another in terms of their COD, both may show a similar efficiency (CODD) when comparing their ability to maintain speed when turning with respect to their linear sprint [15]. A study of rugby players found that faster athletes were less effective at CODs, as their faster approach speed impeded optimal turn execution [53]. Similarly, Clarke et al. suggested that linear velocity has the most significant relationship with COD180 performance (r = 0.71 to 0.76; *p* < 0.01) [54]. On the other hand, a study of young football players found a link between a higher body weight and a reduced ability to execute a COD efficiently (r = −0.65; *p* < 0.01) [55]. These descriptive data provide a useful benchmark for future research, facilitating comparisons between the profiles of young futsal players and allowing for a more accurate analysis of the factors influencing their performance and COD ability.

These descriptive data not only provide a useful benchmark for future research, but also offer relevant insights from a preventive perspective. Although no significant differences were found among playing positions in interlimb asymmetries, all the values remained below the 10% threshold for the unilateral CMJ, horizontal jump, and change-of-direction tests. Maintaining interlimb asymmetries below 10–15% has been suggested as a reasonable threshold [56], as greater asymmetries are associated with an increased injury risk and unilateral overload [57]. These findings support the importance of continuous individual monitoring, as it allows for the identification of athletes who exceed normative values and may benefit from preventive interventions, even in the absence of group-level differences [58].

Similarly, lower CODD values suggest a more efficient braking and turning capacity, which may protect athletes during rapid deceleration actions—a common mechanism in non-contact lower limb injuries [59]. Studies in futsal have identified that multiple sprints with frequent CODs are key contributors to non-contact knee injuries, highlighting the critical role of deceleration quality [60]. Moreover, research on change-of-direction and agility training in youth soccer and futsal indicates that incorporating COD technique drills, eccentric strength work, and multi-component warm-ups (e.g., modified FIFA 11+) not only enhance performance, but also significantly reduce the injury incidence [61]. Therefore, improving the CODD can not only optimize performance, but also enhance physical resilience in young athletes, bridging the gap between explosive ability and biomechanical safety.

The present study has some limitations that should be considered when interpreting the results. Firstly, no direct assessments of body composition (body fat percentage, lean muscle mass) were included. These variables could have provided a more detailed understanding of the athletes’ physical profiles and allowed for a more accurate comparison among playing positions in terms of performance-related morphology. Secondly, important physiological components such as the aerobic capacity and intermittent endurance were not evaluated, despite being fundamental to futsal performance. Including these variables in future research would offer a more holistic view of player characteristics and support the development of individualized, position-specific training strategies.

## 5. Conclusions

In conclusion, this study provides a comprehensive overview of the anthropometric and performance profiles of elite youth futsal players, highlighting both the general characteristics and specific differences among playing positions. Pivots and goalkeepers showed a greater height and body mass, while wingers performed better in speed and change-of-direction tests. Additionally, lower CODD values were associated with a greater efficiency in directional changes, emphasizing the relevance of the acceleration and deceleration capacity in game situations. Although no significant differences were found among positions in terms of interlimb asymmetries, their individual analysis remains essential to identify relevant deviations that could compromise performance or increase the injury risk. These findings reinforce the importance of considering the specific physical demands of each position when planning training and contribute to the development of more precise positional benchmarks in youth futsal.

## Figures and Tables

**Table 1:** Physical and performance characteristics of elite youth male futsal players by playing position (mean ± SD).

Variable	Winger—Left (n = 16)	Winger—Right (n = 27)	Pivot (n = 18)	Goalkeeper (n = 15)	Defender (n = 22)	Total (n = 98)
Age (y) *	17 ± 1	17 ± 1 *	18 ± 1	18 ± 1	17 ± 1	17 ± 1
Height (cm) *	174 ± 5 *^#^	175 ± 6 *^#^	179 ± 5	182 ± 6	175 ± 6 *^#^	177 ± 6
Body mass (kg) *	68 ± 7 ^#^	66 ± 6 *^#^	73 ± 4	78 ± 10	71 ± 7 ^#^	71 ± 8
CMJL (cm)	19.5 ± 2.4	18.6 ± 3.1	18.7 ± 2.2	17.1 ± 2.9	19.4 ± 3.2	18.7 ± 2.9
CMJR (cm)	18.9 ± 3.6	18.3 ± 2.9	18.9 ± 2.7	17.2 ± 2.9	18.8 ± 2.7	18.5 ± 2.9
CMJ Asy (%)	9.8 ± 11.8	8.9 ± 5.1	7.2 ± 4.3	7.3 ± 6.1	7 ± 5.2	8.1 ± 6.6
CMJ (cm)	36.4 ± 3.5	35.0 ± 4.6	36.9 ± 4.2	34.2 ± 4.06	36.9 ± 2.9	35.9 ± 3.9
HJL (cm)	183.9 ± 9.9	184.4 ± 13.2	186.0 ± 10.7	180.5 ± 13.3	186.5 ± 9.4	184.5 ± 11.4
HJR (cm)	183.4 ± 10.5	183.2 ± 9.8	185.4 ± 11.6	181.5 ± 14.4	183.5 ± 9.3	183.4 ± 10.8
HJ Asy (%)	2.4 ± 2.3	4.4 ± 3.3	3.5 ± 2.1	2.7 ± 3	4.6 ± 4.1	3.7 ± 3.2
HJ (cm)	204.6 ± 16.3	206.3 ± 13	210.7 ± 11.4	203.2 ± 13.6	205.6 ± 11.6	206.2 ± 13.1
CODL (s) *	2.58 ± 0.1 ^#^	2.62 ± 0.1 ^#^	2.61 ± 0.1 ^#^	2.76 ± 0.1	2.63 ± 0.1 ^#^	2.64 ± 0.1
CODR (s) *	2.58 ± 0.1 ^#^	2.60 ± 0.1	2.62 ± 0.1	2.71 ± 0.1	2.63 ± 0.1	2.63 ± 0.1
COD Asy	2.6 ± 2.5	3.7 ± 2.9	3.7 ± 2.4	3.6 ± 2.6	2.2 ± 1.9	3.1 ± 2.5
V-cut (s) *	6.94 ± 0.2 ^#^	7.01 ± 0.2	7.03 ± 0.2	7.17 ± 0.2	7.07 ± 0.2	7.04 ± 0.2
10 m (s)	1.88 ± 0.1	1.90 ± 0.1	1.88 ± 0.1	1.93 ± 0.1	1.89 ± 0.1	1.9 ± 0.1
25 m (s)	3.82 ± 0.1	3.83 ± 0.1	3.83 ± 0.1	3.92 ± 0.13	3.84 ± 0.1	3.8 ± 0.1
CODDL (%)	37.2 ± 5.1	38.1 ± 6.8	38.4 ± 5.8	42.7 ± 5.61	39.3 ± 5.9	38.9 ± 6.1
CODDR (%)	37.2 ± 4.9	37.3 ± 6.8	39.1 ± 6.9	39.8 ± 5.94	39.5 ± 6.3	38.5 ± 6.3
CODD25 (%)	81.9 ± 6.8	83.0 ± 6.1	83.8 ± 7.9	82.4 ± 5.45	84.2 ± 8.2	83.2 ± 6.9

CMJR: unilateral countermovement jump with right leg; CMJL: unilateral countermovement jump with left leg; HJR: unilateral horizontal jump with right leg; HJL: unilateral horizontal jump with left leg; COD180R and COD180L: 10 m shuttle sprint with one change of direction to right or left; CMJ: bilateral countermovement jump; HJ: bilateral horizontal jump; 10 m: linear sprint of 10 m; 25 m: linear sprint of 25 m; CODR and CODL: 10 m shuttle sprint with one change of direction of 180° to right or left; CODDL, CODDR, and CODD25: change-of-direction deficit with right or left and V-cut; Asy: asymmetry; * *p* < 0.05 vs. pivot; ^#^ *p* < 0.05 vs. GK.

## Data Availability

Data supporting the findings of this research are available from the corresponding author (Elena Mainer-Pardos) upon reasonable request.

## Abbreviations

The following abbreviations are used in this manuscript:

CMJ	Counter Movement Jump
COD	Change of Direction
CODD	Change-of-Direction Deficit
CV	Coefficient of Variation
ES	Effect Size
HJ	Horizontal Jump
ICC	Intraclass Correlation Coefficient
